# Efficacy of a New Educational Tool to Improve Handrubbing Technique amongst Healthcare Workers: A Controlled, Before-After Study

**DOI:** 10.1371/journal.pone.0105866

**Published:** 2014-09-02

**Authors:** Andrew J. Stewardson, Anne Iten, Véronique Camus, Angèle Gayet-Ageron, Darren Caulfield, Gerard Lacey, Didier Pittet

**Affiliations:** 1 Infection Control Program and World Health Organization Collaborating Centre on Patient Safety, University of Geneva Hospitals and Faculty of Medicine, Geneva, Switzerland; 2 Division of Clinical Epidemiology, University of Geneva Hospitals and Faculty of Medicine, Geneva, Switzerland; 3 Glanta Ltd, Dublin, Ireland; 4 School of Computer Science and Statistics, Trinity College Dublin, Dublin, Ireland; University of Calgary, Canada

## Abstract

**Introduction:**

Hand hygiene is a key component of infection control in healthcare. WHO recommends that healthcare workers perform six specific poses during each hand hygiene action. SureWash (Glanta Ltd, Dublin, Ireland) is a novel device that uses video-measurement technology and immediate feedback to teach this technique. We assessed the impact of self-directed SureWash use on healthcare worker hand hygiene technique and evaluated the device's diagnostic capacity.

**Methods:**

A controlled before-after study: subjects in Group A were exposed to the SureWash for four weeks followed by Group B for 12 weeks. Each subject's hand hygiene technique was assessed by blinded observers at baseline (T_0_) and following intervention periods (T_1_ and T_2_). Primary outcome was performance of a complete hand hygiene action, requiring all six poses during an action lasting ≥20 seconds. The number of poses per hand hygiene action (maximum 6) was assessed in a *post-hoc* analysis. SureWash's diagnostic capacity compared to human observers was assessed using ROC curve analysis.

**Results:**

Thirty-four and 29 healthcare workers were recruited to groups A and B, respectively. No participants performed a complete action at baseline. At T_1_, one Group A participant and no Group B participants performed a complete action. At baseline, the median number of poses performed per action was 2.0 and 1.0 in Groups A and B, respectively (p = 0.12). At T_1_, the number of poses per action was greater in Group A (post-intervention) than Group B (control): median 3.8 and 2.0, respectively (p<0.001). In Group A, the number of poses performed twelve weeks post-intervention (median 3.0) remained higher than baseline (p<0.001). The area under the ROC curves for the 6 poses ranged from 0.59 to 0.88.

**Discussion:**

While no impact on complete actions was demonstrated, SureWash significantly increased the number of poses per hand hygiene action and demonstrated good diagnostic capacity.

## Introduction

Hand hygiene is widely regarded as the single most important intervention to reduce the burden of health care-associated infections and the transmission of antimicrobial resistance within the hospital setting [1]. The contemporary approach to promotion of hand hygiene amongst healthcare workers involves a multimodal strategy incorporating the use of alcohol-based handrub at the point of care [1], [2]. The WHO ‘My 5 Moments for Hand Hygiene’ methodology defines *when* healthcare workers should perform hand hygiene during patient care [3], [4]. Healthcare worker compliance with these indications is part of routine performance feedback, an essential strategy for behaviour change [5]. WHO recommendations also exist for *how* to perform hand hygiene, but these are rarely monitored or included in performance feedback programs. This technique is based on European standards (EN 1500) and involves six distinct steps, or poses [1]. Correct performance of this technique results in increased product coverage and greater reductions of bacterial colony forming units when compared with incomplete actions [6], [7].

SureWash (Glanta Ltd, Dublin, Ireland) is a commercially available device that combines e-learning and patented video measurement technology to teach healthcare workers how to perform a hand hygiene action. It uses interactive on-screen feedback to encourage grounded cognition and reflection on the technique of hand hygiene. The aim of this approach of situated cognition is that the physical act of hand hygiene becomes a prompt to the actions of good technique. The device can be left in a clinical area to be used independently by healthcare workers, and provides immediate and individualised performance feedback.

The primary objective of this study was to assess the efficacy of SureWash to improve hand hygiene technique amongst healthcare workers in an institution with a long history of hand hygiene promotion [2]. Our secondary objective was to evaluate the ability of SureWash to assess the adequacy of hand hygiene actions performed by healthcare worker staff compared to assessment by trained human observers.

## Methods

### Ethics statement

We followed the principles expressed in the Declaration of Helsinki. This study was approved by the Ethics Commission for Human Research at the University of Geneva (protocol 12–258).

### Design

**Figure 1:**
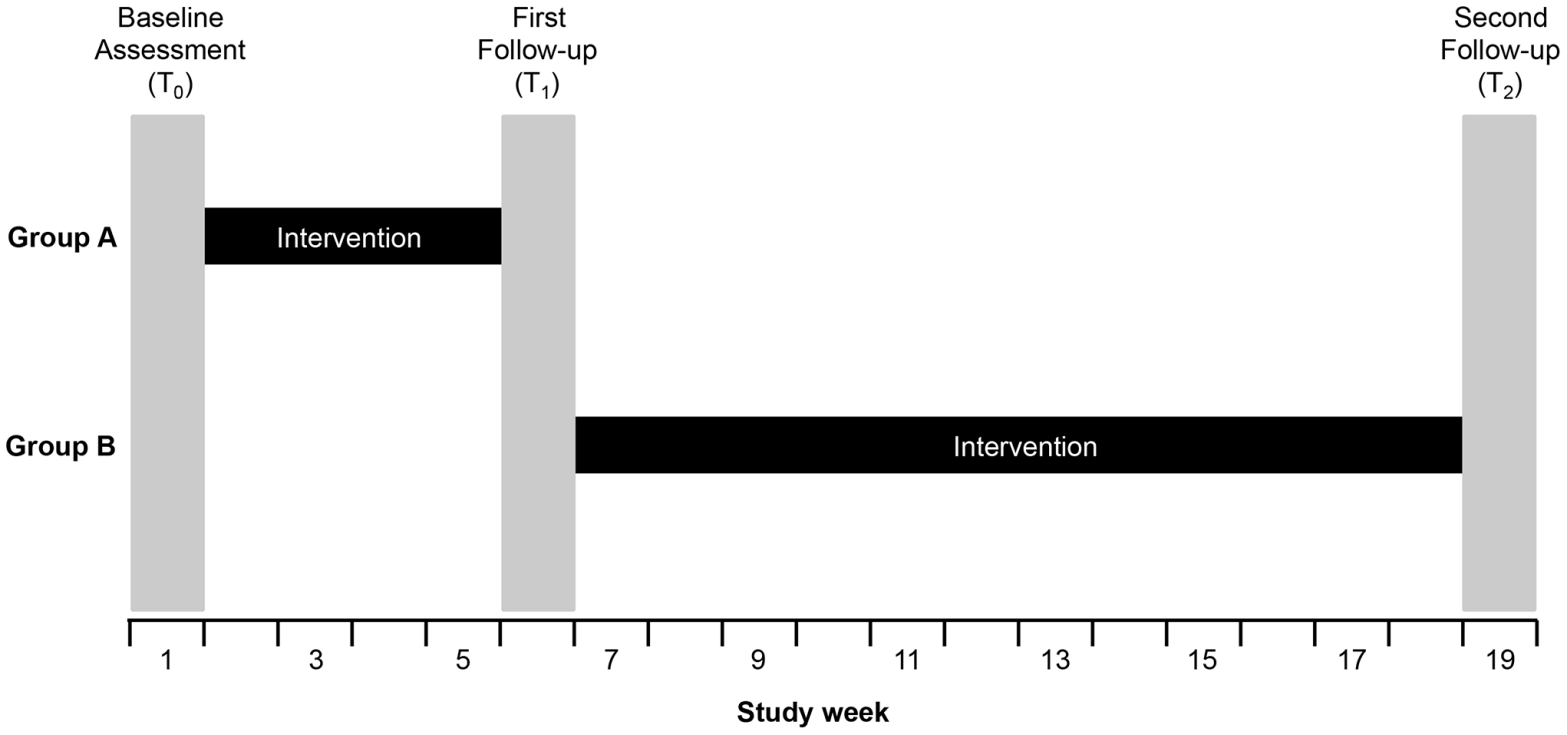
Study design.

We performed a controlled before-after study with blinding of assessors (**Figure 1**). Allocation was not randomised and there was no placebo intervention. First, baseline assessment (T_0_) of hand hygiene technique was performed in two healthcare worker groups (A and B) to ensure that they did not have significantly different pre-intervention hand hygiene technique. The first follow-up (T_1_) assessment was then performed after Group A (intervention group) had received the intervention but Group B (control group) had not. Subsequently, Group B was exposed to the intervention, and a second follow-up (T_2_) measurement of both groups was performed. The application of the intervention to Group B (the original control group) and T_2_ measurement was performed to 1) examine the persistence post-intervention of any improvement in Group A technique, 2) demonstrate reproducibility of intervention effect in two groups of subjects, and 3) allow Group B to benefit from this quality improvement intervention. This design has been referred to as the “untreated-control group design that uses dependent pretest and posttest samples and switching replications” [8].

### Setting

The University of Geneva Hospitals is a 2200-bed primary and tertiary care hospital in Geneva, Switzerland with a long history of hand hygiene promotion [2], [9]. Healthcare workers are exposed to training in hand hygiene technique during an infection control education session on employment commencement, posters throughout the hospital, and guidelines on the infection control intranet site. This study was performed in four acute care wards within the department of internal medicine, consisting of two pairs of adjacent wards on different hospital floors. In 2013, healthcare worker compliance with indications for when to perform hand hygiene was 75.8% (95% confidence interval [CI], 70.7–80.4) as measured by direct observation according to WHO methodology [3], [4].

### Participants

All healthcare workers with patient-care responsibilities in the four participating acute-care wards were eligible to participate on a voluntary basis. Subjects were required to provide written, informed consent and were excluded if 1) unlikely to remain in the study wards throughout the study period, or 2) if they currently worked – or were likely to work during the study period – in wards in both study groups. Healthcare workers that were not recruited were able to use the SureWash during the intervention phase, but were not monitored for the study.

### Intervention

The intervention involved self-directed use of the SureWash unit, which was left unsupervised in the staff tea room. Healthcare workers were able to use it in both ‘training mode’ and ‘assessment mode’ throughout the intervention phase (four weeks in Group A and 12 weeks in Group B). ‘Training mode’ consisted of a slideshow with information regarding when and how to perform hand hygiene, and required healthcare workers to practice their own technique in the presence of immediate feedback. ‘Assessment mode’ involves healthcare workers performing a hand hygiene action and receiving a score (in percentage format) reflecting degree to which each pose was performed correctly and for adequate duration.

### Procedure and data collection

The study was implemented from March to September 2013. The study design is presented in **Figure 1**. At baseline (T_0_), participants completed a brief survey including age, sex, profession, number of years spent working at HUG, and prior participation in the institutional infection control training course. They were then invited by the investigators to perform a hand hygiene action as recommended by hospital guidelines using alcohol-based handrub. This action was recorded by the SureWash device in a purpose-built “study mode” whereby it captured video of the hand hygiene action, but provided no feedback other than to indicate to the user that their hands were in the correct position. The action was assessed by the device and the video stored for subsequent assessment by observers. Immediately after recording this action, each healthcare worker was asked to mime the 6 poses by following an on-screen demonstration.

Following the one-week recruitment and baseline assessment period (T_0_), Group A was exposed to the intervention for four weeks. During this time, the SureWash unit was left in the staff tea room, available for self-directed hand hygiene technique education and training (as described in the intervention section above) at healthcare workers' convenience. Each participant was able to use the device according to their interest and availability: there was no minimum or maximum number of uses required.

Subsequently, during the first follow-up (T_1_), each participant was again asked to perform a hand hygiene action as at T_0_. Group B was then exposed to the intervention for twelve weeks followed by a final assessment of participants in both Group A and B (T_2_).The intervention phase was longer in Group B because whereas the two wards in Group A shared a common tea room, those in Group B did not. In addition, Group B was exposed to the intervention during the summer period when healthcare workers take leave and are therefore frequently absent. Both factors translated to decreased exposure of Group B subjects to the intervention.

**Figure 2:**
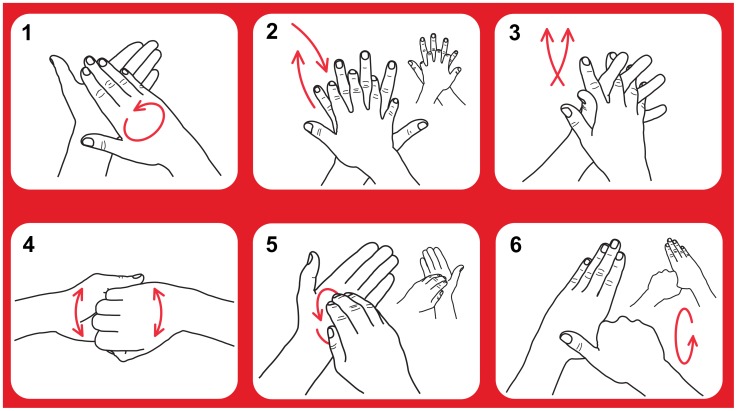
Poses recommended for hand hygiene actions. After applying a palmful of the product in a cupped hand; 1) rub hands palm to palm; 2) right palm over left dorsum with interlaced fingers and vice versa; 3) palm to palm with fingers interlaced; 4) backs of fingers to opposing palms with fingers interlocked; 5) rotational rubbing, backwards and forwards with clasped fingers of right hand in left palm and vice versa; 6) rotational rubbing of left thumb clasped in right palm and vice versa. Text adapted from reference 1.

Following each of the three assessments, two observers (AS and VC) independently reviewed the hand hygiene videos in random order, assessing duration and performance of each pose (**Figure 2**). A purpose-built interface was developed to facilitate this review process. For bilateral poses, performance of each side was assessed separately. The observers were blinded to study group and the SureWash assessment of the action. Following the review process, data could be exported from the device for analysis. This dataset included the following information for every pose: study group, date, the SureWash unit's automatic assessment of pose performance (measure of “effort”) and both reviewers' binary assessment of whether the pose was performed correctly.

### Outcomes

#### Primary objective

The predefined outcome used to assess the impact of the SureWash unit on hand hygiene technique was performance of a complete hand hygiene action as rated by both observers. A hand hygiene action was judged as complete if all six recommended poses were performed and the action lasted for 20 seconds or more (**Figure 2**) [1]. For bilateral poses, both sides had to be performed in order for the posed to be accepted as correctly performed. Poses could be performed in any sequence. The number of times that the SureWash unit was used by each group was recorded as a process measure.

#### Secondary objective

To evaluate the diagnostic capacity of the SureWash unit we compared the human observer assessment (dichotomous) with the SureWash automated assessment (continuous). The SureWash unit produces a measure of the “effort” with which each pose is performed. This “effort” measure was a unit-less continuous variable.

### Statistical methods

The sample size calculation was based on the proportion of healthcare workers in each study group performing complete hand hygiene actions at the first follow-up (T_1_). In the absence of prior data, we estimated that 60% of healthcare professionals would perform a complete hand hygiene action at baseline, and proposed that an absolute improvement of 30% following the SureWash intervention would be clinically pertinent. With a two-sided alpha of 0.05 and a power of 0.8, we required 38 participants in each arm. At baseline, however, we noted that no healthcare workers performed a complete hand hygiene action. We had recruited 34 and 29 subjects into the two groups. We therefore performed an estimation of study power based on this new information. Assuming a 10% loss to follow-up (30 and 26 subjects), we had a power of 0.80 to detect a delta of 30% using two sided alpha of 0.05.

Categorical baseline covariates were presented using counts and percentages, with subjects from the two groups compared using Fisher's exact test. Inter-rater agreement between the two blinded observers was computed using Cohen's kappa. These values were interpreted according to Fleiss [10].

#### Primary objective

The proportion of healthcare workers performing a complete hand hygiene action in each group was compared at T_0_ to assess the assumption that the two groups had similar baseline hand hygiene technique. We then evaluated change in the proportion of healthcare workers performing a complete hand hygiene action from T_0_ to T_1_ in both Group A (intervention) and Group B (control). The initial control group was then exposed to the intervention, and we evaluated change in the proportion of healthcare workers performing a complete hand hygiene action from T_1_ to T_2_ in both Group B (now intervention) and Group A (now control). For each comparison, the null hypothesis of no difference between the two groups was tested using Fisher's exact test.

#### Secondary objective

We used the subset of poses for which the two human observers provided the same assessment. We used Receiver Operating Characteristic (ROC) curve analysis to assess the diagnostic performance of this measure, summarised using area under the curve (AUC). The cutoff value for the SureWash “effort” measure that best discriminated between adequate and inadequate performance (as determined by the human raters) was determined independently for each pose. These optimal cutoffs were selected as the value that maximised Youden's J statistic. We described performance of the device using these optimal cutoffs by presenting sensitivity, specificity, positive predictive value, negative predictive value and accuracy when compared to the human observer. Accuracy is calculated as the number of poses correctly judged by the SureWash device as either adequate or inadequate divided by the total number of poses. The other parameters were calculated as usual. Confidence intervals (CIs) were computed using the Clopper-Pearson method [11].

Statistical analyses were performed using the R software/environment, version 3.0.1 (R Foundation for Statistical Computing), including ‘irr’ and ‘ROCR’ packages [12], [13].

## Results

**Figure 3:**
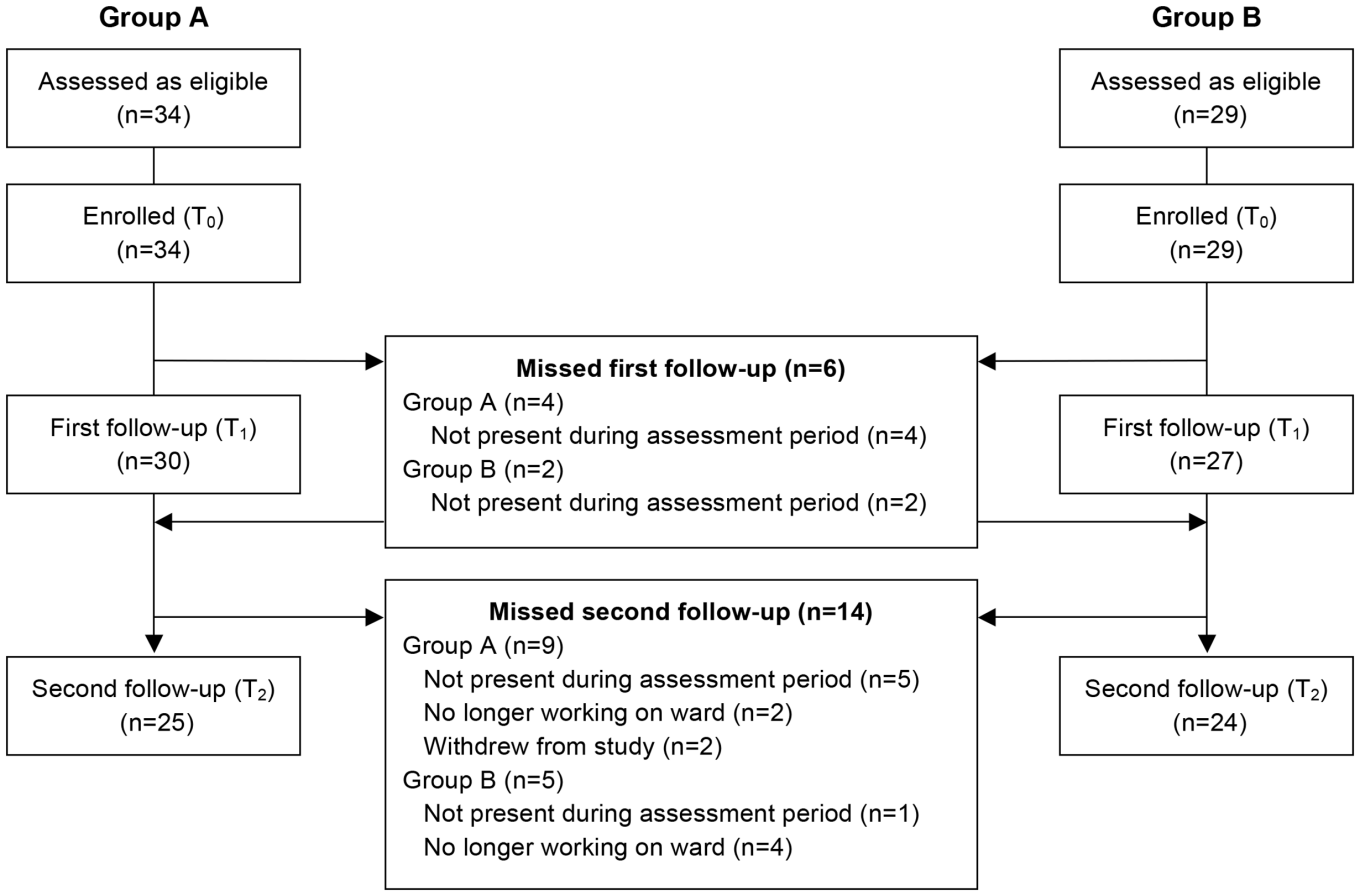
Study flow diagram. All eligible subjects agreed to participate.

Sixty-three healthcare workers were recruited, 34 in Group A and 29 in Group B. No eligible healthcare workers refused to participate (due to scheduled rotations only one doctor was eligible), producing a 100% participation rate. Baseline characteristics are presented in **Table 1**. Follow-up was incomplete. Details of follow-up and reasons for missed data are outlined in the flow diagram (**Figure 3**). Data were missing for six subjects at T_1_ and 14 subjects as T_2_. Two subjects refused to participate on three occasions and were therefore classified as having withdrawn from the trial. The SureWash unit was used 213 and 151 times by healthcare workers in Group A and Group B, respectively, during their intervention phases.

**Table 1:** Baseline characteristics of study participants.

	Group A (n = 34)	Group B (n = 29)	p-value
Female gender	26 (76)	23 (85)	0.522
Age category			0.022
<20	0 (0)	0 (0)	
20–29	1 (4)	4 (20)	
30–39	16 (64)	5 (25)	
40–49	8 (32)	11 (55)	
Profession			0.574
Nurse assistant	10 (29)	5 (19)	
Nurse	21 (62)	19 (70)	
Doctor	1 (3)	0 (0)	
Other	2 (6)	3 (11)	
Years worked at HUG*			0.338
<1	1 (3)	0 (0)	
1–5	4 (13)	4 (15)	
6–10	7 (22)	2 (7)	
>10	20 (63)	21 (78)	
Infection control course completed			0.347
No	9 (26)	12 (44)	
Yes, in 2013	0 (0)	0 (0)	
Yes, in 2012	3 (9)	1 (4)	
Yes, before 2012	22 (65)	14 (52)	

Counts are presented with percentages in parentheses. Responses to each question may not sum to total number of participants due to unanswered questions.

*HUG, University of Geneva Hospitals.

### Primary outcome: Impact of intervention on hand hygiene technique

Agreement between the two human raters for each pose is presented in **Table 2**. According to Fleiss's qualitative descriptors for kappa values, agreement was “fair to good” for poses 1 and 3, and “excellent” for the other four poses.

**Table 2:** Pass rate and interrater agreement between the two human observers regarding performance of each pose.

Pose	Kappa	Descriptor
1	0.735	Fair to good
2 (left/right)	0.974/0.950	Excellent/Excellent
3	0.586	Fair to good
4	0.776	Excellent
5 (left/right)	0.817/0.807	Excellent/Excellent
6 (left/right)	0.813/0.773	Excellent/Excellent

All kappa values were computed using 169 subjects, and were significant, with p-values computed as <0.001. Poses are illustrated in Figure 2.

The primary outcome measure was performance of a complete hand hygiene action. No participants performed a complete hand hygiene action at baseline (T_0_): 0/34 (0.0% [95% CI; 0.0%, 10.3%]) and 0/29 (0.0% [95% CI; 0.0%, 11.9%]) in Groups A and B, respectively. The two groups were therefore similar at baseline (p>0.99).

Between T_0_ and T_1_, Group A received the intervention and Group B acted as control (**Figure 1**). The number of Group A participants that performed a complete action increased from 0/34 (0.0% [95% CI; 0.0%, 10.3%]) at T_0_ to 1/30 (3.3% [95% CI; 0.1%, 17.2%]) at T_1_ (p = 0.47). There was no change in Group B at T_1_, as none of 27 participants (0.0% [95% CI; 0.0%, 12.8%]) performed a complete action (p>0.99).

Between T_1_ and T_2_, Group B received the intervention and Group A acted as control (**Figure 1**). No Group B participants performed a complete action post-intervention 0/24 (0.0% [95% CI; 0.0%, 14.3%]) at T_2_ compared to 0/27 (0.0% [95% CI; 0.0%, 12.8%]) at T_1_ (p>0.99). There was also no change in Group A: 1/30 (3.3% [95% CI; 0.1%, 17.2%]) at T_1_ to 1/25 (4.0% [95% CI; 0.1%, 20.3%]) at T_2_ (p>0.99).

### *Post-hoc* analysis: Impact of intervention on hand hygiene poses per action

Given the rarity of this primary outcome, we performed a post-hoc assessment of the number of poses performed correctly during each hand hygiene action. The rationale for this post-hoc analysis was that not all incomplete actions are equal: an incomplete action with one pose performed is likely to be less effective in removing organisms from the hand than an incomplete action with five poses, for example. Therefore it is of interest to evaluate the impact of the intervention on the number of poses performed per action, as it is plausible that such an effect may have a positive impact on quality of care. However, as a *post-hoc* analysis, these results should be considered exploratory.

First, the proportion of subjects performing each pose, stratified by intervention status, is presented as a descriptive result in **Table 3**. Prior to exposure to the intervention, all poses except pose 2 were performed by less than half of the subject. Pose 4 was performed least frequently. At baseline, healthcare workers generally performed a hand hygiene action comprising of a continuous movement, with infrequent distinct and repeated poses. Improvements were observed in all six poses in the post-intervention period.

**Table 3:** Number of poses performed correctly according to the two observers, stratified by subject intervention status.

Pose	Pre-intervention		Post-intervention	
	Observer 1	Observer 2	Observer 1	Observer 2
1	24 (39.3%)	26 (42.6%)	43 (79.6%)	43 (79.6%)
2 (Left)	40 (65.6%)	39 (63.9%)	40 (74.1%)	41 (75.9%)
2 (Right)	37 (60.7%)	36 (59.0%)	38 (70.4%)	39 (72.2%)
3	23 (37.7%)	23 (37.7%)	44 (81.5%)	37 (68.5%)
4	9 (14.8%)	4 (6.6%)	18 (33.3%)	11 (20.4%)
5 (Left)	21 (34.4%)	16 (26.2%)	31 (57.4%)	30 (55.6%)
5 (Right)	21 (34.4%)	17 (27.9%)	31 (57.4%)	31 (57.4%)
6 (Left)	11 (18.0%)	7 (11.5%)	22 (40.7%)	23 (42.6%)
6 (Right)	11 (18.0%)	5 (8.2%)	22 (40.7%)	25 (46.3%)

“Pre-intervention” includes Group A subjects at T_0_ and Group B subjects at T_1_ (n = 61). “Post-intervention” includes Group A subjects at T_1_ and Group B subjects at T_2_ (n = 54). Poses are illustrated in Figure 2.

**Figure 4:**
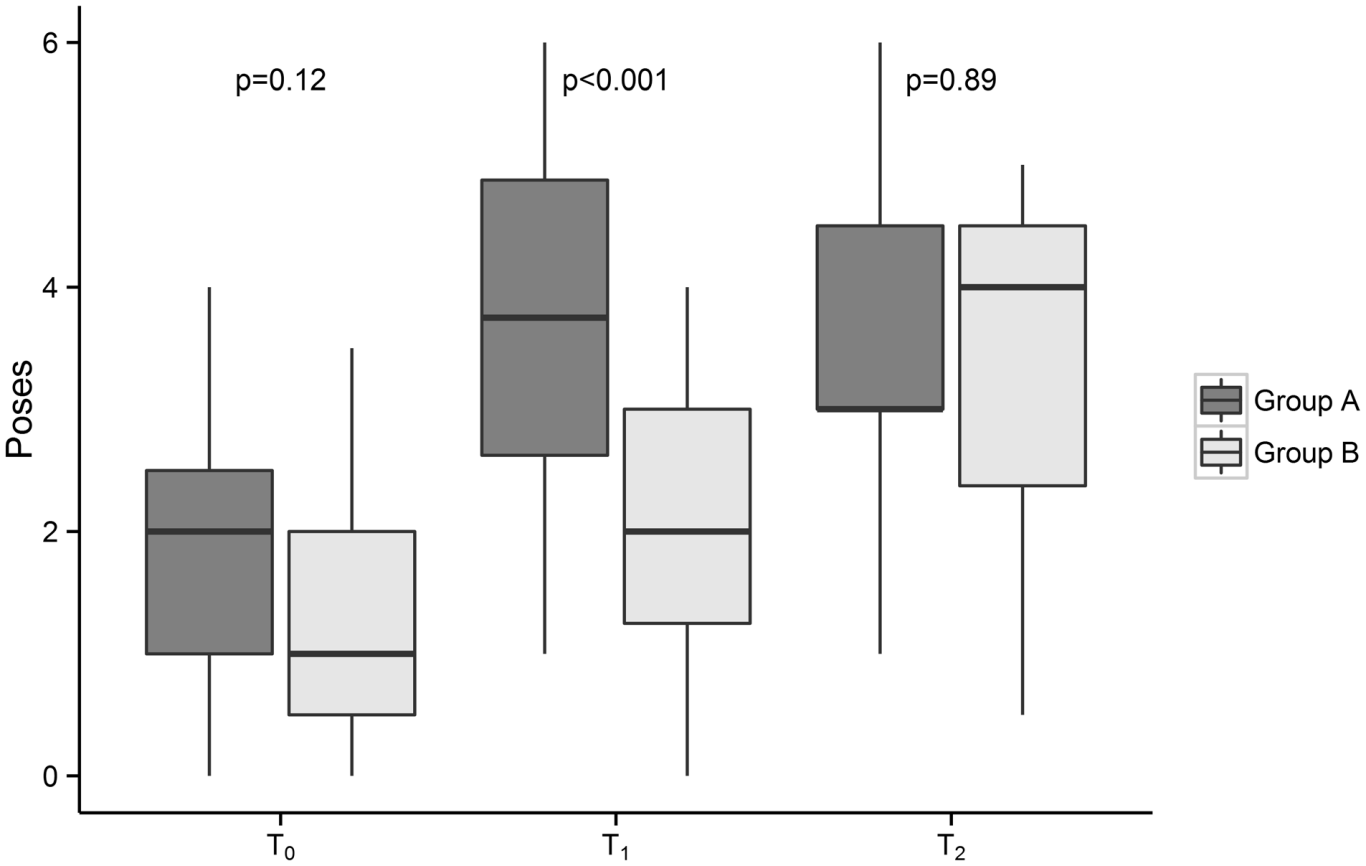
Number of poses performed correctly per hand hygiene action, by study group and study phase. Group A was exposed to the intervention for four weeks between baseline (T_0_) and the first follow-up (T_1_). Group B was exposed to the invention for 12 weeks between the first follow-up (T_1_) and the second follow-up (T_2_). Median and interquartile ranges are represented by the horizontal line and box, respectively. Upper and lower whiskers extend to minimum and maximum values that lie within 1.5 times the interquartile range from the 75th and 25th percentile, respectively. Each p-value relates to the null hypothesis that the two groups perform the same number of poses correctly at that time point.

Second, the number of poses performed per action by the two groups were compared (**Figure 4** **)**. The two groups performed a similar number of poses correctly at T_0_, before either had been exposed to the intervention: median 2.0 (IQR, 1.5) in Group A and median 1.0 (IQR, 1.5) in Group B (p = 0.12).

The number of poses performed by Group A (intervention) subjects increased from median 2.0 (IQR, 1.5) at T_0_ to 3.8 (IQR, 2.3) immediately post-intervention at T_1_ (p<0.001). Over the same period, there was a lesser absolute increase in the number of poses performed by Group B (control) subjects: median 1.0 (IQR, 1.5) at T_0_ to 2.0 (IQR, 1.8) at T_1_ (p = 0.03). At T_1_, Group A performed more poses that Group B (p<0.001).

Group B was then exposed to the intervention. The number of poses performed by Group B subjects increased from median 2.0 (IQR, 1.8) at T_1_ to 4.0 (IQR, 2.1) at T_2_ (p<0.001). Over the same period, there was no significant change in the number of poses performed by Group A (now control) subjects: median 3.8 (IQR, 2.3) at T_1_ to 3.0 (IQR, 1.5) at T_2_ (p = 0.49). The number of poses performed by Group A subjects at T_2_ remained significantly higher than baseline (p<0.001). At T_2_, Group A and Group B subjects performed a similar number of poses per action (p = 0.89).

### Secondary outcome: Diagnostic capacity of SureWash

**Figure 5:**
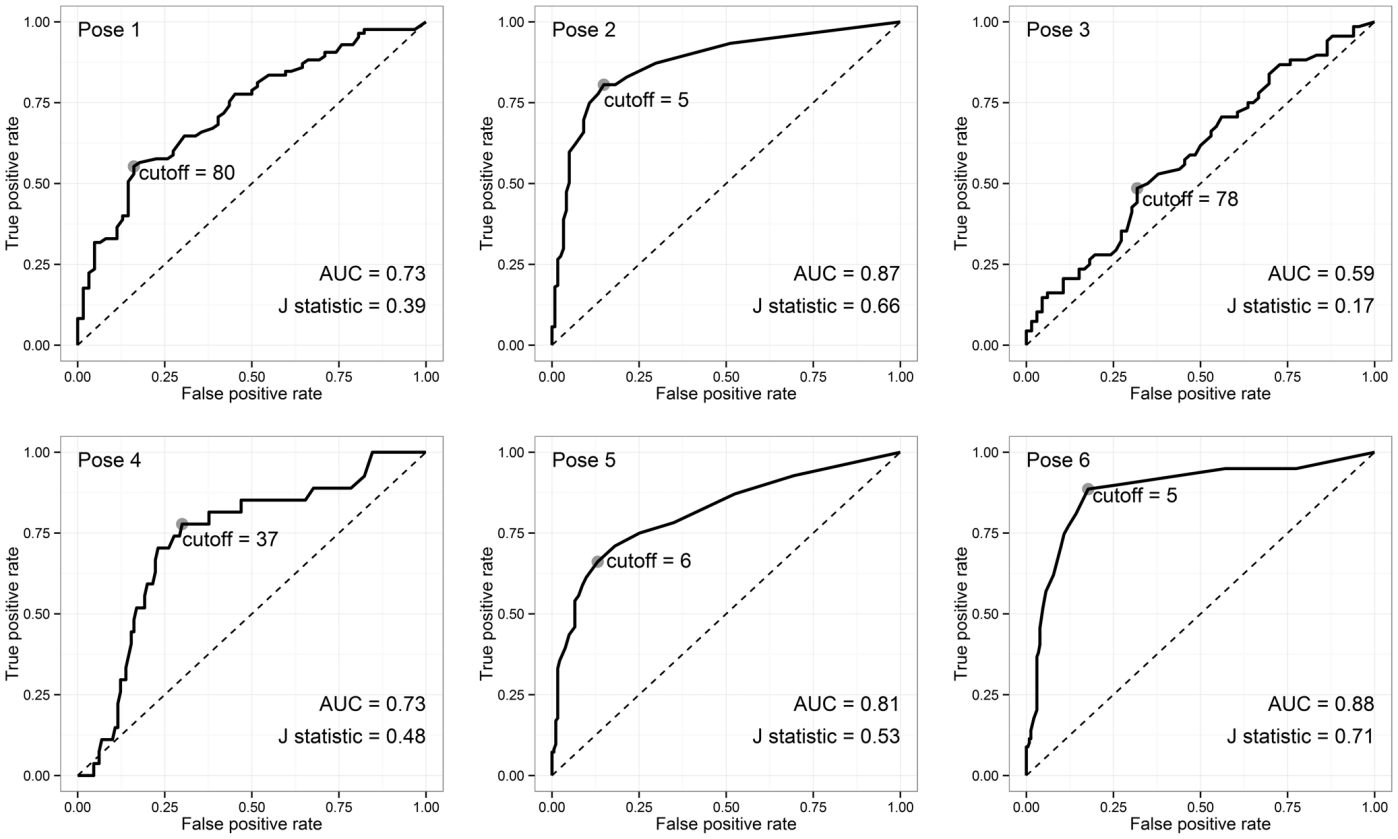
Receiver operating characteristic curves for each pose. Grey points indicate the diagnostic cutoff that maximises Youden's J statistic. AUC, area under the curve.

ROC curves for each pose are presented in **Figure 5**. Performance characteristics when the optimal cutoff (which maximised Youden's J statistic) was employed are presented in **Table 4**.

**Table 4:** Performance characteristics of SureWash as a diagnostic test when compared to human observers.

Pose	n	Sensitivity	Specificity	Accuracy	PPV	NPV
Pose 1	147	0.55 (0.44–0.66)	0.84 (0.72–0.92)	0.67 (0.59–0.75)	0.82 (0.7–0.91)	0.58 (0.47–0.68)
Pose 2	332	0.81 (0.75–0.86)	0.85 (0.78–0.91)	0.82 (0.78–0.86)	0.90 (0.85–0.94)	0.72 (0.63–0.79)
Pose 3	134	0.49 (0.36–0.61)	0.68 (0.56–0.79)	0.58 (0.49–0.67)	0.61 (0.47–0.74)	0.56 (0.45–0.67)
Pose 4	157	0.78 (0.58–0.91)	0.70 (0.61–0.78)	0.71 (0.64–0.78)	0.35 (0.23–0.48)	0.94 (0.87–0.98)
Pose 5	307	0.66 (0.57–0.74)	0.87 (0.81–0.91)	0.79 (0.73–0.83)	0.77 (0.68–0.85)	0.79 (0.73–0.85)
Pose 6	310	0.89 (0.79–0.95)	0.82 (0.77–0.87)	0.84 (0.79–0.88)	0.63 (0.53–0.72)	0.95 (0.92–0.98)

Computed using cutoff values selected to maximise Youden's J statistic. Estimations provided with 95% confidence intervals in parentheses. Poses are illustrated in Figure 2.

PPV, positive predictive value; NPV, negative predictive value.

## Discussion

This trial was performed to assess the utility of SureWash in improving hand rubbing technique in a healthcare institution with a long history of hand hygiene promotion [2], [9]. Baseline (T_0_) results demonstrated a need for such an intervention, with no healthcare workers able to perform a hand hygiene action as recommended by WHO [14]. This trial did not demonstrate an impact of the SureWash device on the proportion of healthcare workers able to perform a complete hand hygiene action using strict criteria. However, a *post-hoc* analysis demonstrated that exposure to this device had a significant and (in Group A) durable impact on the number of poses performed correctly per hand hygiene action. Finally, the device demonstrated good diagnostic capacity when compared to human observers.

These findings are consistent with and extend those of two previous publications using the SureWash unit. Gosh *et al.* used the device in one clinical ward during two six-day phases; the first without feedback (16 subjects) and the second with feedback (34 subjects) [15]. Inter-rater agreement between two human observers was 0.76 (Krippendof's alpha), with agreement of 0.74 and 0.56 for each observer with the device. Using a less strict definition of complete hand hygiene action (1 second for each pose), the pass rate for hand hygiene actions increased modestly from 62.5% to 64.7% (p<0.05). Higgins *et al.* used the SureWash device as part of an institution-wide multimodal hand hygiene promotion campaign [16]. The pass rate for handwashing (rather than hand rubbing) technique using adenosine triphosphate monitoring increased from 52% before implementation of training with SureWash to 79% after (p<0.001). Compliance with the six recommended poses was not specifically assessed. Our study confirms the diagnostic capacity of SureWash using a larger sample size that Gosh *et al.* and builds on data from both studies regarding its impact on hand hygiene technique by using a controlled study design implemented in the absence of concurrent interventions, with assessment of each pose, and by using hand rubbing, the preferred technique for routine hand hygiene [1].

The importance of hand hygiene technique with regard to product coverage and reduction in bacterial counts on hands has been demonstrated previously [6], [7], [17]. The baseline results of this trial suggests that an infection control course on employment commencement, educational posters in clinical areas and availability of guidelines are not sufficient to teach hand hygiene technique. Monitoring and performance feedback is a key strategy to improving healthcare worker hand hygiene behaviour [1], [5], but this traditionally focuses on *when* to perform it rather than *how*. More intensive training can be resource intensive [7]. For example, in a recent study using UV-light technology to assess hand hygiene technique immediately following training, 5200 healthcare workers were exposed to 15-minute education sessions in groups of five to eight [18]. This was a major logistic operation and required at least 160 hours work. In contrast, a potential strength of the SureWash unit is that it can be left in clinical areas for independent use by healthcare workers, liberating infection control professionals for other activities. This benefit needs to be counter-weighed against the operating cost of the device.

We did not demonstrate an impact on the number of healthcare workers performing a complete hand hygiene action. In fact, only two such actions were observed during the study. This may reflect the stringency of the outcome measure definition: six poses (three of which must be repeated bilaterally) performed correctly during at least 20 seconds as judged by two independent human raters. We would consider “poses per action” or a microbiologic measure a preferable outcome measure when designing future studies. However this result belies a change in behaviour that occurred nevertheless. At baseline, when asked to perform a hand hygiene action, the overwhelming majority of healthcare workers slid one hand over the other in a continuous, seemingly random movement. Following the intervention, we observed that participants instead made repeated stereotyped poses. This can be appreciated in the *post-hoc* analysis of poses per action. Several approaches could be considered to optimise the impact of this intervention in busy clinical settings: alternative placement or longer exposure to the device; ward based role-models or ‘champions’ to inspire friendly competition, benchmarking of results against other wards; or a more formal credentialing requirement. Uptake is likely to vary between settings, and a flexible approach involving frontline ownership may be most effective.

This trial demonstrates that SureWash has good capacity to distinguish between correctly and incorrectly performed poses. However, two issues should be considered when reviewing these data. First, this analysis was performed on recordings made during the three assessment periods (in “study mode”), when immediate feedback was not provided to healthcare workers. During standard use, healthcare workers receive immediate feedback in the form of green bars that extend when the pose is being correctly performed. Thus healthcare workers quickly refine their technique by making minor adjustments to hand position or movement, and agreement between the device and human observers could be expected to increase. Second, though good inter-rater agreement between the two observers supports their reliability as a reference diagnostic technique, the human review process was clearly imperfect, involving a degree of subjective judgement.

These data must be interpreted in the context of the study design. First, this trial was designed to assess the efficacy of SureWash as an educational tool. Consequently, we assessed healthcare workers' capacity to perform hand hygiene technique on request, rather than covertly monitoring actual hand hygiene technique during routine patient care. Second, this trial does not provide data regarding the importance of performing hand hygiene as per WHO recommendations. However, the superiority of the WHO technique with regard to product coverage and reduction in bacterial colony forming units has been demonstrated previously [7]. Third, human review of video images was used to assess the primary outcome and also as “gold-standard” reference test to evaluate the device's diagnostic performance (secondary objective). Whilst we attempted to quantify reliability of human observers by presenting inter-rater agreement, we acknowledge that this “gold standard” is imperfect. Finally, due to anonymity considerations, we were unable to track individual healthcare workers' performance through each of the three assessments and correlate improvement with their use of the SureWash unit.

In summary, no healthcare workers were able to perform a complete hand hygiene action at baseline despite a long institutional history of hand hygiene promotion. While we were unable to demonstrate an increase in complete hand hygiene actions, exploratory *post-hoc* analysis suggested that exposure to SureWash significantly increased the number of poses performed per action, and this effect persisted 12 weeks post intervention. This study identifies a need for further study of hand hygiene technique and demonstrates the potential utility of the SureWash device. Future studies should explore methods to maximise the uptake and effectiveness of this device as well as the impact of improved hand hygiene technique on transmission events or laboratory surrogates.
